# Co-infection with Cbp^+^ *Streptococcus mutans* and *Candida albicans* is associated with root caries in older adults

**DOI:** 10.1080/20002297.2026.2667029

**Published:** 2026-05-05

**Authors:** Bruna Albuquerque Garcia, Louise Morais Dornelas-Figueira, C. Katrak, Luiz Fernando Wurdig Roesch, Scott Lance Tomar, Grace Adams, Dayane Oliveira, Marcelle Matos Nascimento, Jacqueline Abranches

**Affiliations:** a Department of Restorative Dental Science, University of Florida College of Dentistry, Gainesville, FL, USA; b Department of Oral Biology, University of Florida College of Dentistry, Gainesville, FL, USA; c Comprehensive Care and Allied Professions Department, Indiana University School of Dentistry, Indianapolis, IN, USA; d Microbiology & Cell Science Department, University of Florida Institute of Food and Agricultural Sciences, Gainesville, FL, USA; e Department of Population Oral Health, University of Illinois Chicago College of Dentistry, Chicago, IL, USA; f Department of Restorative Dentistry, University of Buffalo School of Dental Medicine, Buffalo, NY, USA

**Keywords:** Dental caries, root caries, caries risk assessment, caries risk factors, oral microbiome, Streptococcus mutans, Candida, collagen binding proteins

## Abstract

Background: Gingival recession increases with age, exposing root surfaces and raising susceptibility to root caries. As the global population ages, root caries prevalence is expected to rise, underscoring the need to better understand its risk factors. Objective: To investigate the association between co-infection with Cbp^+^ *Streptococcus mutans* and *Candida albicans* and root caries in older adults with gingival recession, while characterizing the root plaque microbiome and evaluating multifactorial risk factors. Design: This cross-sectional study included 117 adults ≥65 years; 56 with root caries and 61 without. Saliva and site-specific supragingival plaque samples were collected to assess Mutans streptococci and *Candida* spp., along with microbiome composition. Demographic, behavioral, biological, dental, and medical factors were also evaluated. Results: Participants with root caries had higher salivary levels of Mutans streptococci and *Candida* spp. (p = 0.0122 and p = 0.0013). Co-infection with Cbp^+^ *S. mutans* and *C. albicans* was significantly associated with root caries (p = 0.0003). Microbiome analysis showed enrichment of *Capnocytophaga leadbetteri* in diseased root surfaces. Individuals with root caries were more likely to report xerostomia and less likely to use an electric toothbrush or floss daily. Conclusions: These findings highlight microbial and behavioral factors associated with root caries, offering insights into potential prevention and management strategies.

## Introduction

Root caries lesions are located on the root surfaces of teeth, affecting both the cementum and dentin. They are most commonly associated with gingival recession, which exposes root surfaces and makes them more susceptible to the caries process [1]. As people age, gingival recession becomes more prevalent [2], increasing the risk of root caries, particularly among older adults [3]. According to the 2017–2020 National Health and Nutrition Examination Survey (NHANES), approximately 12.5% of adults aged 60 years or older in the United States were affected by root caries [4]. With the global population aging rapidly and the number of root caries cases expected to rise, it is crucial to gain a deeper understanding of the risk factors associated with this condition.

The aetiology of root caries is similar to that of coronal caries, as both are multifactorial diseases of a polymicrobial nature that are influenced by biological, behavioural and environmental factors [5]. However, the substrates in which these lesions develop differ considerably. Compared with enamel that covers coronal tooth surfaces, which is primarily composed of minerals, root surfaces consist of dentin and cementum, which are tissues with lower mineral content and a higher organic component, particularly collagen [6]. Due to these structural differences, biological factors such as microbial composition, metabolic activity and interspecies interactions associated with coronal caries may differ from those involved in root caries.

Among the biological factors, the metabolic activity of cariogenic microorganisms plays a key role in the initiation and progression of dental caries [7]. *Streptococcus mutans* is a Gram-positive, facultative anaerobic bacterium that is highly cariogenic due to its ability to metabolise sugars into organic acids such as lactic acid, produce an extracellular polysaccharide-rich biofilm and thrive in acidic environments, ultimately leading to demineralisation of the enamel and cementum, resulting in cavity formation [8]. While the overall detection of *S. mutans* in saliva is not a strong predictor of caries risk [9], evidence suggests that the presence of *S. mutans* strains harbouring bona fide collagen-binding proteins (Cbps), specifically Cnm or Cbm, is associated with increased caries risk [10]. Cnm and Cbm are found in nearly 20% of *S. mutans* clinical isolates [10,11]. Although these proteins are not universally required for the cariogenicity of *S. mutans*, studies have shown that their presence contributes to *S. mutans* colonisation by mediating strong adhesion to collagen [12].

Another important microorganism involved in the pathogenesis of coronal and root caries is the opportunistic fungal pathobiont *Candida albicans* [13–15]. In fact, *C. albicans* synergistically interacts with *S. mutans,* and co-infection with these two microorganisms is commonly associated with severe caries in children and also with root caries [15–20]. This relationship is supported by animal studies demonstrating that co-infection with *S. mutans* and *C. albicans* elicits more extensive caries than infection with either microorganism individually [21]. Several factors contribute to this synergism in caries severity: *C. albicans* can utilise lactic acid excreted by *S. mutans* as an energy source, it is highly acidogenic and aciduric, can reduce the oxygen tension of oral biofilms and protects against oxidative stress [22,23]. In turn, glucosyltransferase B (GtfB) secreted by *S. mutans* can bind to the surface mannans of *C. albicans,* allowing the yeast to synthesise adhesive *α-*glucan polysaccharides in the presence of sucrose and further enhance the formation of a cariogenic biofilm matrix [24,25]. Of note*, C. albicans* also efficiently binds to collagenous surfaces [26] and possesses collagenase activity [27,28] that can also contribute to dentinal caries risk and progression [28].

Based on the evidence that *C. albicans* and the Cbps of *S. mutans* facilitate adhesion to collagen-rich surfaces like dental roots and that co-infection with *S. mutans* and *C. albicans* promotes the formation of highly cariogenic biofilms, we conducted a cross-sectional study to investigate whether co-infection with Cbp-positive (Cbp^+^) *S. mutans* and *C. albicans* is associated with root caries in older adults with gingival recession. We also, as a secondary objective of this study, compared the microbiome composition of subjects with and without root caries to identify potential biomarkers of healthy and diseased root plaque.

Furthermore, recognising that disease risk is multifactorial and best evaluated using multivariable risk assessment models rather than single predictors, we also examined a range of potential contributing factors. These included demographic characteristics (age, sex, race/ethnicity), behavioural factors (dietary risk, fluoride exposure, oral hygiene practices, tobacco and drug use), biological indicators (salivary and plaque microbiome, dry mouth), dental history (caries experience, periodontal status, presence of restorations or crowns, use of complete or partial dentures) and medical history (hypertension, diabetes, heart disease and radiation or chemotherapy).

## Materials and methods

### Recruitment and enrolment

This cross-sectional clinical study was reviewed and approved by the Institutional Review Board of the University of Florida (IRB202100916 and IRB202202059). All methods were performed following STROBE guidelines and regulations. Eligible participants were adults aged 65 years or older with at least one tooth presenting an exposed root surface. Exclusion criteria included: (i) use of antibiotics within the past month, (ii) use of immunosuppressants at the time of the study visit and (iii) use of antimicrobial mouthwashes within the past month. Recruitment took place between December 2022 and June 2023 from a convenience sample during routine dental visits at the predoctoral dental clinics of the University of Florida College of Dentistry. A power analysis was performed using the Sample Size—Proportions (Standard: Specify P₀) procedure with the Chi-squared statistic, based on data from our previous clinical study [10]. The analysis indicated that a minimum of 104 adults were required to adequately address the study aims, assuming a statistical power of 80% and a significance level of *α* = 0.05. A total of 120 participants were enroled. Written informed consent was obtained from all participants before clinical examination and sample collection, and participants were informed of their right to withdraw from the study at any time. Clinical evaluations were conducted by calibrated dentists. Caries detection and diagnosis followed the International Caries Detection and Assessment System II (ICDAS-II) visual criteria [29] and the Lesion Activity Assessment (LAA) scoring system [30]. Based on oral health status, participants were classified as either healthy (H) or having root caries (R). Participants in the H group presented with at least one exposed root surface but no active carious lesions (ICDAS caries and root caries score 0), while participants in the R group presented with at least one non-carious, exposed root surface (ICDAS root caries score 0) and at least one exposed root surface with an active, cavitated root caries lesion (ICDAS root caries score 2). Lesion activity was determined by assessing clinical appearance, plaque stagnation and tactile sensation.

### Data source

The following data were extracted from participants' electronic medical and dental records: (a) Demographic characteristics: age, sex and race/ethnicity; (b) Medical history: presence of hypertension (yes or no), history of radiation or chemotherapy (yes or no), heart disease (yes or no), diabetes (no diabetes, controlled diabetes or uncontrolled diabetes), drug use (no, marijuana or other) and tobacco use (no, past or current); (c) Dental history: presence of existing root surface restorations, number of existing crowns, presence of complete or removable partial dentures, number of decayed, missing (due to caries) or filled teeth (DMFT) index, percent of teeth with of gum recession, time since last dental visit; (d) Periodontal status (healthy, gingivitis, periodontitis stage 1/2, periodontitis stage 3/4 or history of periodontitis); and (e) Caries risk and protective factors: dietary risk (low, medium or high), daily fluoride exposure, presence of dry mouth (yes or no self-reported), brushing frequency (not daily, once a day or twice a day), toothbrush type (manual or electric), flossing frequency (not daily or daily) and caries risk levels (low, medium, high or extreme).

### Specimen collection

**Figure 1.**
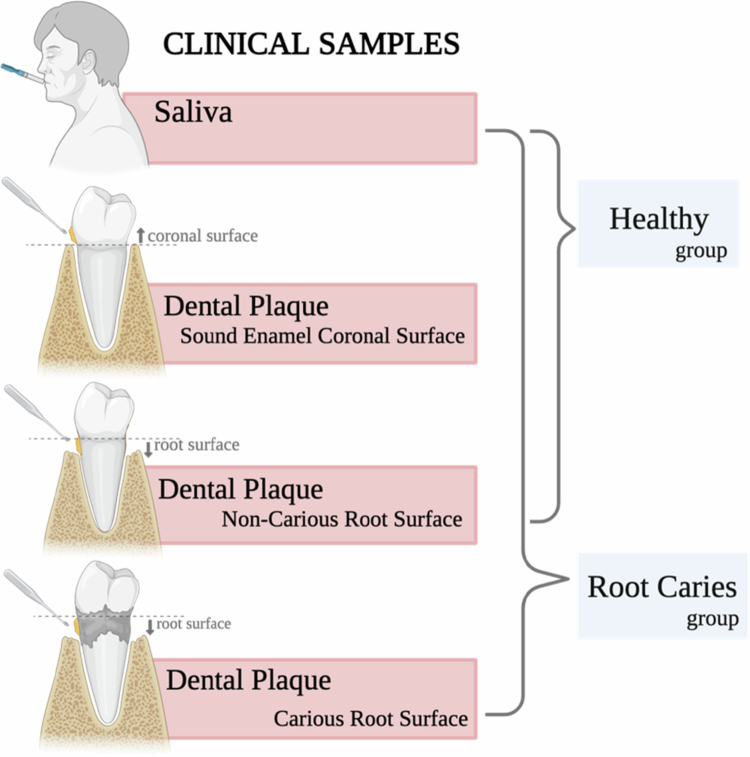
Clinical sample collection workflow. Saliva was collected from participants in the healthy (H) and root caries (R) groups using sterile straws and tubes. Supragingival plaque was obtained with a sterile periodontal curette from sound enamel surfaces (E) and non-carious root surfaces (NC) in both groups and from carious root surfaces (RC) exclusively in the R group.

Saliva and supragingival plaque samples were collected from all participants at various tooth sites, as shown in Figure 1. Approximately 5 ml of whole, non-stimulated saliva (S) was collected using sterile straws and plastic tubes from all participants. Supragingival plaque was collected from sound enamel surfaces (E) and non-carious root surfaces (NC) from participants in both the H and R groups. Additionally, plaque from a carious root surface (RC) was collected exclusively from individuals in the R group. All plaque samples were collected using a sterile periodontal curette and deposited into a 1 ml sterile phosphate-buffered saline solution tube and kept on ice until sample processing.

### Microbiological data

Within three hours of specimen collection, the samples were processed by an investigator masked to the root caries status of the participants.

To quantify *Candida* spp. infection levels, serial dilutions of saliva and plaque were plated onto Sabouraud agar (Becton, Dickinson and Company BBL™, Franklin Lakes, NJ, USA) supplemented with 0.4 mg/ml chloramphenicol (SAB). Plaque counts were normalised to total plaque protein content determined by bicinchoninic acid protein (BCA) assay. Later, eight *Candida* spp. colonies were streaked on Hardy CHROM agar (HardyCHROM™, Santa Maria, CA, USA), a chromogenic agar plate, for identification of *Candida* species (*C. albicans, C. tropicalis, C. glabrata and C. krusei*). On CHROMagar Candida, green colonies may represent either *C. albicans* or *C. dubliniensis*. Because chromogenic agar does not reliably distinguish these species, we classified all green colonies as *C. albicans*, given its markedly higher prevalence in the oral cavity. We acknowledge that few isolates may have been *C. dubliniensis* and therefore results referring to ‘*C. albicans*’ should be interpreted as pertaining to the *C. albicans/C. dubliniensis* complex (herein referred to as *Candida albicans*), and this constitutes a limitation of the study. Of note, *C. glabrata and C. krusei* are now referred to as *Nakaseomyces glabratus* and *Pichia kudriavzevii,* respectively.

To quantify and isolate Mutans streptococci, serially diluted specimens were plated on Difco Mitis Salivarius agar (Becton, Dickinson and Company BBL™, Franklin Lakes, NJ, USA) supplemented with 0.388 mg/ml bacitracin (MSB). The MSB plates were incubated for 48 hours at 37 °C with 5% CO_2_. Mutans streptococci plaque counts were normalised to total plaque protein by BCA assays. Eight S. *mutans*-like colonies from each site of collection (S, E, NC and RC) were selected from the MSB plates and streaked onto tryptic soy agar plates (Becton, Dickinson and Company BBL™, Franklin Lakes, NJ, USA). Colonies were boiled for 10 min in 100 μl of water to kill the cells and release DNA. Then, 3–5 μl of the boiled samples were used as a template in polymerase chain reactions (PCR) with *S. mutans*-specific primers [31] for *S. mutans* identification (Supplementary Table S1.) The presence of genes coding for the collagen- and laminin-binding proteins, *cnm* and *cbm*, was also assessed in the confirmed *S. mutans* colonies. This was done using PCR with *cnm*[32]- and *cbm*[33]*-* specific primers. The laboratory strains UA159 (*cnm*^-^), OMZ175 (*cnm*^+^) and LAR01 (*cbm*^+^) were used as controls. Finally, to assess whether the *cnm*^+^ or *cbm*^+^*S. mutans* isolates were also expressing collagen and laminin-binding proteins, a collagen-binding assay was performed as detailed in [34].

### Microbiome composition

Saliva and plaque samples from 40 randomly selected participants were assessed for microbiome composition by using the Nanopore MinION sequencing platform (Oxford Nanopore Technologies, Oxford, UK) and the RESCUE pipeline to classify bacteria through long-read 16S-ITS-23S [35]. Briefly, the reads were classified using EMU v3.4.1 [36] by mapping against four curated databases as described in Petrone et al. [35]. Saliva and plaque DNA samples were extracted using the Quick-DNATM Fungal/Bacterial Miniprep Kit following the manufacturer's protocol (Zymo Research Corp., Irvine, CA, USA). DNA was quantified using the dsDNA High Sensitivity Assay (Thermo Fisher Scientific, MA, USA) on the Qubit 4.0. Library preparation adhered to the Ligation sequencing DNA V14 (SQK-LSK114, Oxford Nanopore Technologies) protocol, and the final library was loaded onto an R10.4.1 (FLO-MIN106) flow cell and sequenced for 72 hours. The raw nanopore signal was basecalled using Dorado v5.0.0 on the super high-accuracy model, sup. The contingency table, assembled at the bacterial species level, was rarefied to the minimum library size to perform alpha diversity analyses as previously recommended [37]. Alpha diversity was calculated using the vegan package v2.6-6.1 with the Shannon diversity index [38]. The Shapiro‒Wilk test was used to determine the normality of the Shannon diversity index, and the non-parametric Kruskal–Wallis test was implemented to compare the medians among groups. Alpha diversity from the saliva samples and plaque samples was analysed separately. Beta diversity was examined using PCoA with Euclidean distances calculated after centred log-ratio transformation of the bacterial species abundance without rarefaction. Sample groups influencing beta diversity were tested using permutational multivariate analysis of variance (PERMANOVA) through the Adonis function in the vegan package with 999 permutations using the same distance matrix based on CLR-transformed abundance and Euclidean distances. The differential abundance analysis was performed using ALDEx2 without rarefaction as described before [10]. All microbiome bioinformatics analyses were performed in R Version 4.2.3.

### Statistical analysis

The main analytic approach was based on an unmatched case‒control study design. The bivariate association between categorical demographic, clinical and behavioural variables and root caries status was tested by using contingency table analysis and Cochran-Mantel-Haenszel chi-square tests. Interval-level variables were compared between those with root caries and those without root caries by using the unpaired t-test. Variables significantly associated with root caries status in bivariate analysis were tested for inclusion in a parsimonious multivariable logistic regression model. Starting with a full model and using backward stepwise selection, variables that were no longer significantly associated with root caries status were removed from the model. The final model included one variable that had been removed in stepwise selection (the presence of *C. albicans*) to test its independent association because the model included an indicator of co-infection with *S. mutans* and *C. albicans*. All analyses were performed using the SAS version 9.4 software package (SAS Institute, Cary, NC).

The colony-forming unit (CFU) counts of Mutans streptococci and *Candida spp.* in saliva and plaque samples collected from different sites were compared between healthy individuals and those with root caries by using the Mann–Whitney test, followed by Dunn's multiple comparisons test. To accommodate the presence of zero values in the plaque and saliva assays, log transformations with an added constant were applied, for example, log(CFU counts in plaque) = log(CFU counts in plaque + 0.00001). Analyses were performed with GraphPad Prism version 10 (GraphPad Software, San Diego, CA, USA).

## Results

### Enrolment and demographic analysis of study participants

Three participants were excluded from the study due to withdrawal of consent, age and technical issues related to sample collection. Thus, the final analysis included 117 study participants. Of these, 61 were classified as healthy (H) and 56 were classified as having root caries (R).

The mean age of all participants was 73.3 (SD = 6.3) years (Table 1). There was no statistically significant difference in age between the H (mean = 72.7 years) and R groups (mean = 73.8 years; *p* = .3520). Of the 117 participants, 69 (58.9%) were male and 48 (41.1%) were female. Males comprised a significantly larger proportion of the R group (73.2%) than the H group (45.9%; *p* = .0028). Most participants were self-identified as White, non-Hispanic (81.2%), followed by Black, non-Hispanic (8.5%) and there was no significant association between race/ethnicity and root caries status (*p* = .4235).

**Table 1.** Selected demographic characteristics of study participants, by root caries status.

	All participants (*n* = 117) Number (%)	Root caries status		
Characteristic		Healthy (*n* = 61) Number (%)	Root caries (*n* = 56) Number (%)	*P* value
Age in years, mean ± SD	73.3 ± 6.3	72.7 ± 5.9	73.8 ± 6.7	.3520^a^
**Sex**				.0028^b^
Female	48 (41.1)	33 (54.1)	15 (26.7)	
Male	69 (58.9)	28 (45.9)	41 (73.2)	
**Race/ethnicity**				.4235^b^
White, non-Hispanic	95 (81.2)	47 (77.0)	48 (85.7)	
Black or African American, non-Hispanic	10 (8.5)	7 (11.5)	3 (5.3)	
American Indian, non-Hispanic	4 (3.4)	2 (3.2)	2 (3.6)	
Asian	2 (1.7)	0 (0.0)	2 (3.6)	
Hispanic	6 (5.1)	5 (8.2)	1 (1.7)	

Of the 120 participants enroled, three were excluded and the tables reflect data from the remaining 117 participants.

^a^ T-test.

^b^ Cochran-Mantel-Haenszel chi-square.

### Medical and dental histories as possible risk factors for root caries

Participants with root caries did not significantly differ from those without root caries on the prevalence of reported hypertension, history of radiation or chemotherapy, heart disease, diabetes, tobacco use or recreational drug use (Table 2).

**Table 2.** Selected medical history characteristics, by root caries status.

Variable	All participants (*n* = 117) number (%)	Root caries status		
		Healthy (*n* = 61) number (%)	Root caries (*n* = 56) number (%)	*P* value^a^
Hypertension	81 (69.2)	39 (63.9)	42 (75.0)	.1971
History of radiation	11 (9.4)	5 (8.2)	6 (10.7)	.6426
History of chemotherapy	10 (8.5)	4 (6.6)	6 (10.7)	.4237
Heart disease	24 (20.5)	11 (18.0)	13 (23.2)	.4899
Diabetes				.6566
Controlled	27 (23.1)	12 (19.6)	15 (26.7)	
Uncontrolled	2 (1.7)	1 (1.6)	1 (1.8)	
Tobacco use				.4870
Current	3 (2.6)	1 (1.6)	2 (3.6)	
Former	41 (35.0)	19 (31.1)	22 (39.3)	
Drug use	7 (6.0)	3 (4.9)	4 (7.1)	.5752

^a^ Cochran-Mantel-Haenszel chi-square.

Compared to participants without root caries, those with root caries were significantly more likely to have dental root surface restorations (80.3% vs. 40.9%; *p* < .0001) and had significantly higher DMFT (22.4 v. 18.7; *p* = .0001) (Table 3). Complete dentures in one arch were much less prevalent in the R group than in the H group (1.8% vs. 16.3%; *p* = .0071). Periodontal status differed significantly between the two groups (*p* < .0001), with periodontitis much more prevalent among those with R (75.0%) than H (29.5%) participants.

**Table 3.** Select dental and periodontal characteristics by root caries status.

Variable	All participants (*n* = 117) number (%)	Root caries status		
		Healthy (*n* = 61) number (%)	Root caries (*n* = 56) number (%)	*p* value
Presence of root surface restorations	70 (59.8)	25 (40.9)	45 (80.3)	<.0001^b^
Number of existing crowns, mean ± SD	4.4 ± 4.0	4.4 ± 3.6	4.3 ± 4.4	.9252^a^
Presence of a complete denture in only one arch	11 (9.4)	10 (16.3)	1 (1.8)	.0071^b^
Presence of removable partial dentures	22 (18.8)	14 (22.9)	8 (14.2)	.2328^b^
Number of remaining natural teeth, mean ± SD	20.10 ± 5.8	19.77 ± 6.4	20.46 ± 5.1	.8370
DMFT, mean ± SD	20.5 ± 5.4	18.7 ± 5.5	22.4 ± 4.3	.0001^a^
DF, mean ± SD	12.61 ± 5.5	10.5 ± 5.0	14.89 ± 5.0	<.0001^a^
M, mean ± SD	7.89 ± 5.8	8.2 ± 6.4	7.5 ± 5.1	.8370^a^
Percentage of teeth with gum recession, mean ± SD	52.5 ± 21.0	49.1 ± 21.7	56.2 ± 19.7	.0679^a^
Time since last dental visit				.8786^b^
Within the past year	43 (37.7)	21 (35.5)	22 (40)	
1 to <2 years	33 (28.9)	18 (30.5)	15 (27.7)	
2 years or more	38 (33.3)	20 (33.9)	18 (32.7)	
Periodontal status				<.0001^a^
Healthy	2 (1.71)	1 (1.6)	1 (1.8)	
Gingivitis	11 (9.4)	9 (14.7)	2 (3.5)	
Periodontitis stage 1 or 2	18 (15.3)	4 (6.6)	14 (25.0)	
Periodontitis stage 3 or 4	42 (35.9)	14 (22.9)	28 (50.0)	
History of periodontitis	44 (37.6)	33 (54.1)	11 (19.6)	

^a^ T-test.

^b^ Cochran-Mantel-Haenszel chi-square.

As shown in Table 4, participants with root caries were more likely than those without root caries to report having a dry mouth (28.6 vs. 11.4%; *p* = .0206), and less likely to report using an electric toothbrush (21.4 vs. 44.2%; *p* = .0154) or floss daily (48.2 vs. 72.1%; *p* = .0084).

**Table 4.** Caries risk and protective factors, by root caries status.

	All participants (*n* = 117) number (%)	Root caries status		
Factor		Healthy (*n* = 61) number (%)	Root caries (*n* = 56) number (%)	*p* value^a^
**Dietary risk**				.1181
Low	55 (47.0)	35 (57.3)	20 (35.7)	
Med	33 (28.2)	13 (21.3)	20 (35.7)	
High	21 (17.9)	10 (16.3)	11 (19.6)	
Missing	8 (6.8)	3 (4.9)	5 (8.9)	
Daily fluoride exposure	112 (95.7)	60 (98.3)	52 (92.9)	.1432
Dry mouth	23 (19.6)	7 (11.4)	16 (28.6)	.0206
**Brushing frequency**				.0971
Not daily	4 (3.4)	0	4 (7.1)	
1x day	24 (20.5)	12 (19.7)	12 (21.4)	
2x day	89 (76.1)	49 (80.3)	40 (71.4)	
**Toothbrush type**				.0154
Manual	76 (64.9)	34 (55.7)	42 (75.0)	
Electric	39 (33.3)	27 (44.2)	12 (21.4)	
Daily flossing	71 (60.7)	44 (72.1)	27 (48.2)	.0084

^a^ Cochran-Mantel-Haenszel chi-square.

### Investigation of microbiological factors associated with root caries

**Figure 2.**
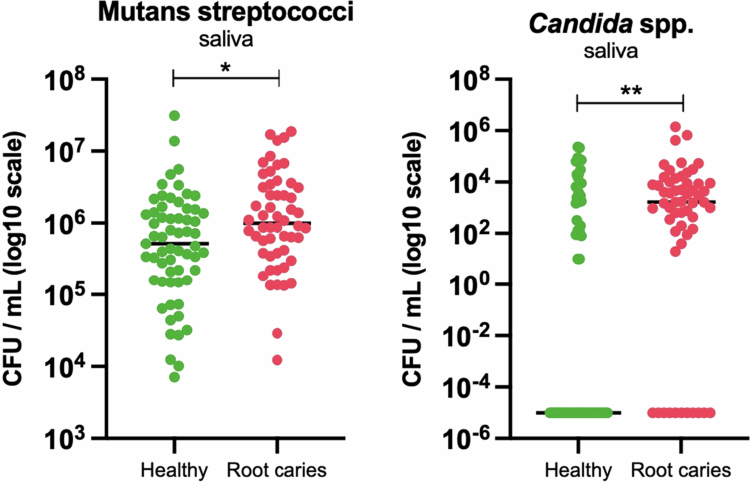
Comparison of Mutans streptococci and *Candida* spp. counts in saliva samples from healthy participants and those with root caries. CFU/mL values were log₁₀-transformed for analysis; tick marks indicate back-transformed values. A pseudocount of 0.00001 was added to all samples to account for zeros prior to transformation. Statistical significance was assessed using Mann–Whiteny test.

**Figure 3.**
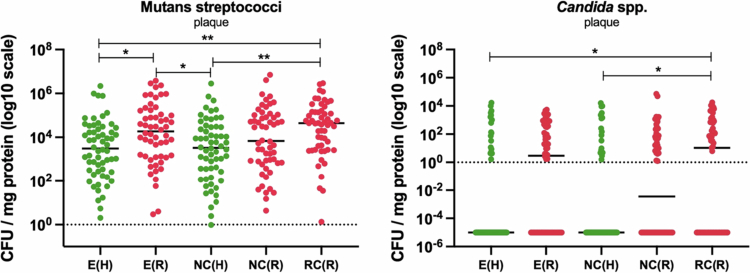
Comparison of infection levels of Mutans streptococci and *Candida* spp. in plaque samples collected from healthy participants and those with root caries. Plaques were collected from enamel (E), non-carious root (NC) and carious root surfaces (RC). CFU/mg protein values were log₁₀-transformed for analysis; tick marks indicate back-transformed values. A pseudocount of 0.00001 was added to all samples to account for zeros prior to transformation. Statistical significance was assessed using Mann–Whiteny and Dunn's multiple comparisons tests.

Given the well-established association of *S. mutans* and *C. albicans* in the aetiology of coronal caries, we assessed their presence and levels by collecting oral samples from all participants to estimate their infection levels in both study groups. Because saliva is considered a potential source of biological markers and is relatively safe and straightforward to collect, we initiated the study with these samples. We found that participants with root caries had significantly higher salivary Mutans streptococci and *Candida* spp. counts compared to healthy participants (*p* = 0.0122 and *p* = 0.0013, respectively) (Figure 2). Recognising that the composition of the oral microbiome is highly site-specific and dependent on the distinctive features of the local substrate, we further analysed supragingival dental plaque samples collected from three different intraoral sites: non-carious enamel surfaces (E), non-carious exposed root surfaces (NC) and carious root surfaces (RC). Carious root site plaque was enriched for both Mutans streptococci (*p* < .01) and Candida spp. (*p* < .05). Enamel plaque from root caries participants also had significantly more Mutans streptococci (*p* < .05) than enamel plaque from healthy participants (Figure 3).

Next, we confirmed the presence of *S. mutans* among the Mutans streptococci isolates by PCR using *S. mutans*-specific primers (Table 5). Then, the identified *S. mutans* strains were also tested by PCR for the presence of the Cbp coding genes *cnm* or *cbm*. To assess functional collagen-binding activity, we also conducted a collagen-binding assay. Overall, the presence of *S. mutans* was detected in 89.7% of participants. There was no statistically significant association between the presence of *S. mutans* and root caries (*p* = .0956). However, a statistically significant association was found when examining the prevalence of *S. mutans* strains harbouring the Cbp genes (*cnm* and *cbm*) (*p* = .0289) and with strong binding to collagen (*p* = .0350). Moreover, we did not find a significant association of the prevalence of Cbp^−^ *S. mutans* with root caries (*p* = .3567).

**Table 5.** Presence of *S. mutans* and *Candida*, by root caries status.

	Root caries status			
Microorganism	All participants (*n* = 117) number (%)	Healthy (*n* = 61) number (%)	Root caries (*n* = 56) number (%)	*p* value^a^
*S. mutans*	105 (89.7)	52 (85.2)	53 (94.6)	.0956
Cbp^+^ *S. mutans* (harbouring genes)	29 (24.8)	10 (16.4)	19 (33.9)	.0289
Cbp^+^ *S. mutans* (binding to collagen)	22 (18.8)	7 (11.5)	15 (26.8)	.0350
Cbp^−^ *S. mutans* (not harbouring genes)	76 (65.0)	42 (68.9)	34 (60.7)	.3567
*Candida* spp.	75 (64.1)	29 (47.5)	46 (82.1)	.0001
*C. albicans*	45 (38.5)	14 (23.0)	31 (55.4)	.0003
More than one species of *Candida*	14 (12.0)	3 (4.9)	11 (19.6)	.0146
Co-infection with *S. mutans* and *Candida* spp.	71 (60.7)	26 (42.6)	45 (80.4)	<.0001
Co-infection with *S. mutans* and *C. albicans*	43 (36.8)	13 (21.3)	30 (53.6)	.0003
Co-infection with Cbp^+^ *S. mutans* (harbouring genes) and *Candida* spp.	19 (16.2)	3 (4.9)	16 (28.6)	.0006
Co-infection with Cbp^+^ *S. mutans* (harbouring genes) and *C. albicans*	11 (9.4)	0 (0.0)	11 (19.6)	.0003
Co-infection with Cbp^+^ *S. mutans* (binding to collagen) and *Candida* spp.	14 (12.0)	2 (3.3)	12 (21.4)	.0026
Co-infection with Cbp^+^ *S. mutans* (binding to collagen) and *C. albicans*	8 (6.8)	0 (0.0)	8 (14.3)	.0023
Co-infection with Cbp^−^ *S. mutans* (not harbouring genes) and *Candida* spp.	52 (44.4)	23 (37.7)	29 (51.8)	.1257
Co-infection with Cbp^−^ *S. mutans* (not harbouring genes) and *C. albicans*	32 (27.4)	13 (21.3)	19 (33.9)	.1261

^a^ Cochran-Mantel-Haenszel chi-square.

For the *Candida* spp. isolates, we determined the prevalence of *C. albicans* and identified multispecies infection using CHROM agar medium. These prevalence estimates were then compared between the two study groups (Table 5). The presence of *Candida* spp., including *C. albicans*, was significantly associated with root caries. Among participants with root caries, 82.1% carried *Candida* spp. compared to 47.5% of those without root caries (*p* = .0001). Similarly, *C. albicans* was detected in 55.4% of participants with root caries, compared to 23.0% of healthy participants (*p* = .0003). Colonisation with more than one species of *Candida* was also significantly associated with root caries (*p* = .0146), indicating that complex *Candida* communities may contribute to the disease process.

### *C. albicans-S. mutans* co-infection significantly increases the risk of root caries

We assessed the prevalence of co-infections involving *Candida* spp. or *C. albicans* with *S. mutans* and Cbp+ and Cbp^−^ *S. mutans* to evaluate their potential role as risk factors for root caries (Table 5). Notably, co-infection with *Candida* spp. and *S. mutans* was found in 80.4% of participants with root caries compared to 42.6% of those without (*p* < .0001). Similarly, *C. albicans-S. mutans* co-infection was detected in 53.6% of participants with root caries compared to 21.3% in the healthy group (*p* = .0003). Co-infection with Cbp^+^ *S. mutans* and *C. albicans* is associated with root caries (*p* = 0.0003). However, we did not find an association of co-infection with Cbp^−^ *S. mutans* and *Candida* spp (*p* = .1257) and of Cbp^−^ *S. mutans* and *Candida albicans* (*p* = .1261) with root caries.

### Plaque microbiome analysis revealed that *Capnocytophaga leadbetteri*is overrepresented in root surfaces in patients with root caries lesions

**Figure 4.**
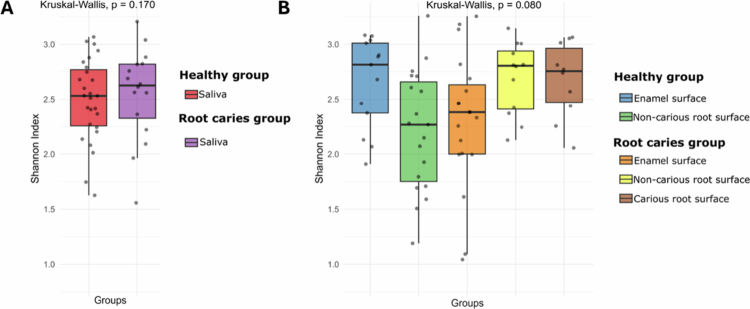
Shannon diversity index of salivary (A) and plaque (B) microbiome in healthy participants and those with root caries. Individual points represent samples, and boxplots show the median and interquartile range. Saliva and plaque samples were analysed separately. No significant difference was found among the groups (*p* > 0.05) according to the non-parametric Kruskal–Wallis.

To further characterise the microbiota associated with root caries and identify potential microbial biomarkers, we investigated the salivary, enamel and root plaque microbiome of 40 randomly selected participants (20 in each group) using the Nanopore MinION platform. Following basecalling, 2,002,298 raw reads were generated, with an N50 read length of 4,458 bases. Quality metrics indicated that 44% of bases achieved Phred quality scores of Q20 or higher. After quality filtering and taxonomy assignment, 100,185 high-quality sequences were retained and classified at the species level. A total of 268 species distributed among 9 phyla were detected across all samples. Firmicutes was the most dominant phylum, constituting 59% of healthy enamel, 67% of healthy root, 71% of root caries, 64% of root caries enamel and 62% of root caries non-carious root. The diversity of microbial species, considering both richness and evenness of saliva and plaque samples, was assessed by using the Shannon diversity index (Figure 4). No statistically significant differences in alpha diversity were observed between sample types or study groups (*p* > .05).

**Figure 5.**
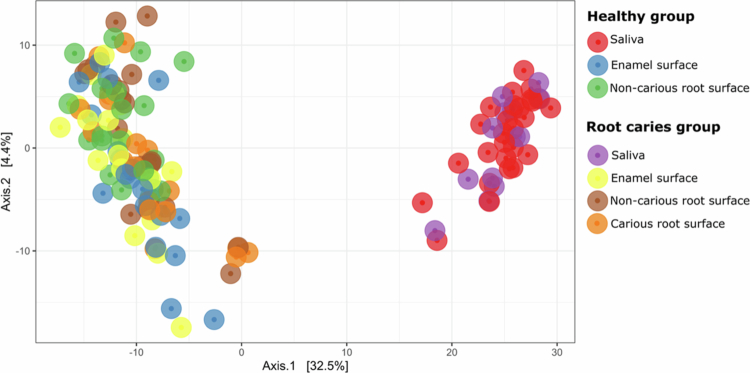
Principal coordinates analysis (PCoA) plots based on Euclidean distances calculated from centred log-ratio (CLR)–transformed species-level abundance data comparing the bacterial species community composition of saliva and plaque samples from healthy participants and those with root caries. Each point represents a sample, and distances between points reflect differences in overall microbial community structure. The percentages shown on Axis 1 and Axis 2 indicate the proportion of total variance in the CLR-transformed community data explained by each principal coordinate. The statistical differences among groups were calculated by PERMANOVA analysis based on 999 permutations. R^2^ and adjusted *p*-values are presented in Supplementary Table S2.

The bacterial community structures among the oral samples were assessed by using Principal Coordinates Analysis (PCoA). As shown in Figure 5, although no apparent differences were detected between the root caries and healthy groups, a clear segregation was observed between saliva and dental plaque samples. As saliva and plaque samples represent two distinct habitats, and we observed statistical differences between these habitats (PERMANOVA R² = 0.31, *p*-value = 0.001), we also performed an individual PCoA analysis for each of these groups (Supplementary Figure S1).

To identify which sample sites were significantly different from each other, we conducted a pairwise PERMANOVA analysis comparing the bacterial community structure of each pair of sample sites separately (Supplementary Table S2). Among individuals without root caries, statistically significant differences were observed between samples from saliva and plaque from enamel (R² = 0.42, *p*-adjusted = .021), and between saliva and plaque from non-carious root surfaces (R² = 0.38, *p*-adjusted = .021). Interestingly, a statistically significant difference was observed when comparing plaque from enamel surfaces with plaque from non-carious root surfaces in individuals without root caries (R² = 0.05, *p*-adjusted = .021), reinforcing the site-specific nature of the oral microbiome. A similar trend was observed among individuals with root caries (see Supplementary Table S2).

When comparing samples collected from similar sites between subjects with and without root caries, no statistically significant differences were observed. Specifically, the microbial community of saliva samples from healthy individuals did not differ from the microbial community of saliva from individuals with root caries (R² = 0.01, *p*-adjusted = 1), nor did plaque from enamel surfaces (R² = 0.03, *p*-adjusted = 1) or plaque from root surfaces (R² = 0.02, *p*-adjusted = 1). These findings suggest that the overall microbial community composition does not differ significantly between individuals with root caries and those without root caries.

**Figure 6.**
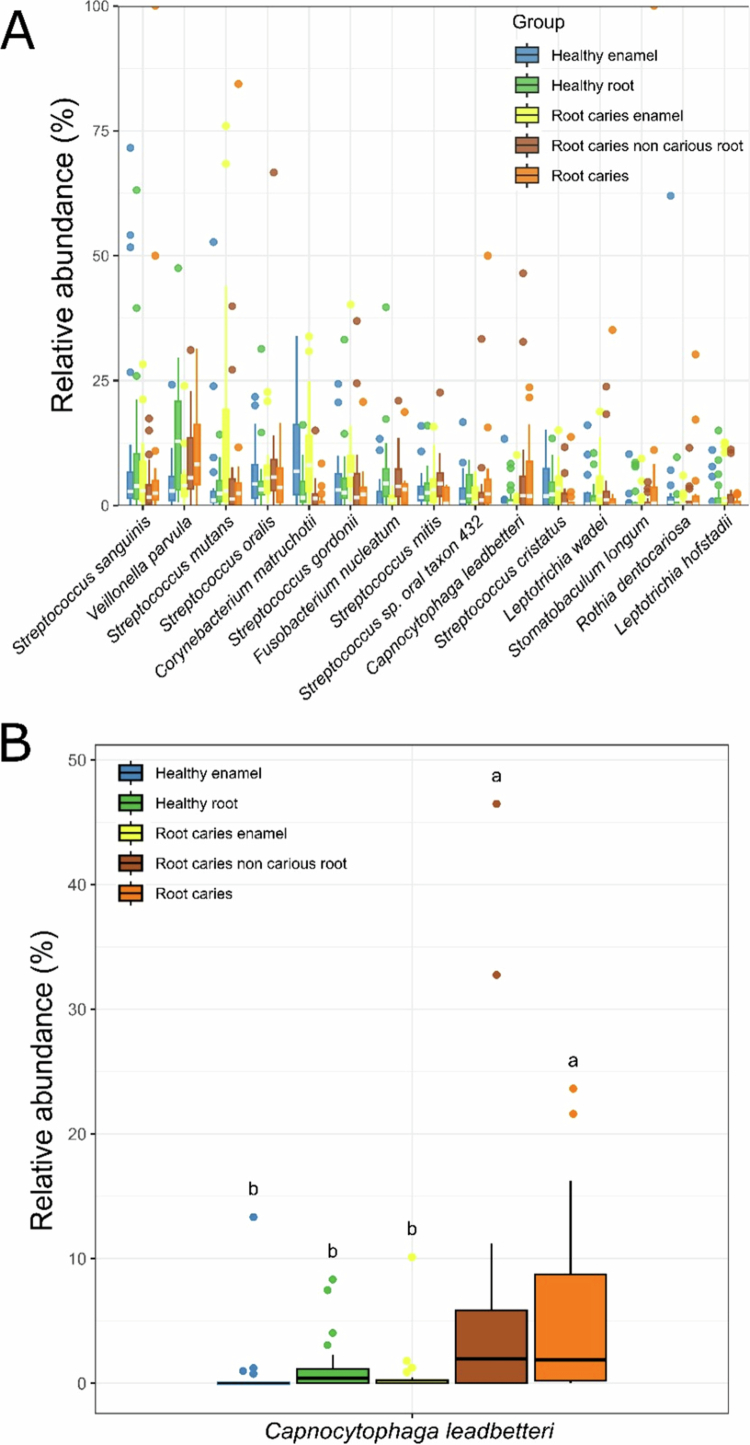
Relative abundance of bacterial species among healthy participants and those with root caries. A) Top 15 most abundant microbes. B) Total abundances of *Capnocytophaga leadbetteri*. Means with the same letter are not significantly different (FDR-adjusted *p* > .05), while different letters are significantly different (FDR *p* < .05).

Next, we evaluated the relative abundance of the 15 most abundant bacterial species in dental and root plaque from both study groups (Figure 6A). Using the ALDEx2 ANOVA-like method, a significantly higher abundance of *Veillonella parvula* was observed in plaque from non-carious (*p* = .02) and carious (*p* = .05) root surfaces compared to plaque from enamel in subjects with root caries. Although not statistically significant, a high abundance of *Veillonella parvula* was also noted in plaque from non-carious root surfaces in healthy subjects compared to plaque from enamel. Interestingly, *Capnocytophaga leadbetteri* (Figure 6B) was significantly more abundant in plaque from non-carious and carious root surfaces of participants with root caries than in plaque from enamel surfaces of participants with root caries, as well as in plaque from all sites in healthy individuals, suggesting its potential as a biomarker for predicting root caries.

### Integration of demographic, microbiological and periodontal factors in predicting root caries development

Unlike many previous studies that have primarily focused either on demographic, medical/dental factors and behavioural habits or solely on the microbiological aspects of root caries, we uniquely integrated all those domains within the same study. This comprehensive approach allows us to explore the interplay between host-related and microbiological factors and their collective impact on root caries development. After identifying variables significantly associated with root caries in bivariate analyses, we constructed a parsimonious logistic regression model to assess their simultaneous association with root caries (Table 6). Based on this model, males in our study sample were three times more likely than females to have root caries (adjusted odds ratio [OR] = 3.30; 95% confidence interval [CI]: 1.31, 12.14). Periodontal status was also a significant correlate of root caries as individuals diagnosed with periodontitis stages I–IV or with a history of periodontitis who presented as clinically healthy with a reduced periodontium (based on the 2017 World Workshop on the Classification of Periodontal Diseases) were more likely to exhibit root caries (adjusted OR = 5.31; 95% CI: 1.16–24.33). The presence of Cbp^+^ *S. mutans* (adjusted odds ratio [OR] = 4.02; 95% confidence interval [CI]: 1.33–12.14) and the presence of *C. albicans* (adjusted OR = 5.88; 95% CI: 2.23–14.97) were each significantly and independently associated with root caries status. Reported use of dental floss less than daily was also a significant independent correlate of root caries (adjusted OR = 2.78; 95% CI: 1.23–7.49). Inclusion of the co-infection term for Cbp^+^ *S. mutans* and *C. albicans* was not included in the multivariable model, due to all 11 participants with co-infection of Cbp^+^ *S. mutans* and *C. albicans* had root caries.

**Table 6.** Final multivariable logistic regression model for demographic, clinical and microbiologic predictors of root caries.

Variable	Crude odds ratio (95% confidence interval)	Adjusted* odds ratio (95% confidence interval)
**Sex**		
Female	Ref	Ref
Male	3.22 (1.48, 7.00)	3.30 (1.31, 12.14)
**Periodontal status**		
Healthy or Gingivitis	Ref	Ref
Periodontitis**	3.46 (0.90, 13.31)	5.31 (1.16, 24.33)
**Flossing frequency**		
Daily	Ref	Ref
Less than daily	2.78 (1.29, 5.99)	3.03 (1.23, 7.49)
Cbp^+^ *S. mutans* presence	2.62 (1.09, 6.28)	4.02 (1.33, 12.14)
*C. albicans* presence	4.16 (1.88, 9.23)	5.88 (2.23, 14.97)

^*^ Final model includes all variables shown in the table. Ref = reference group.

^**^ The category ‘periodontitis’ includes individuals classified with periodontitis stages 1 or 2, periodontitis stages 3 or 4, as well as individuals with a history of periodontitis who presented as clinically healthy with a reduced periodontium.

## Discussion

Clinical studies frequently group coronal and root caries due to shared aetiological factors and overlapping preventive approaches. However, root caries presents distinct characteristics from coronal caries that range from different substrates (cementum/dentin vs. enamel) to differences in microbial composition, which warrant separate investigation. Understanding specific factors associated with root caries is essential for identifying individuals at increased risk and developing targeted preventive strategies. In this study, we evaluated the association between root caries in older adults and a range of potential contributing factors that include microbial, behavioural and clinical factors. Notably, one of the most striking findings was the strong association between root caries and co-infection with *S. mutans*, especially with Cbp^+^ strains and *C. albicans*.

*S. mutans* prevalence and association with caries appear to vary with age. In our previous study [10] involving children aged 6 to 72 months, the presence of *S. mutans* was strongly and significantly associated with early childhood caries (ECC), being detected in 80.0% of children with recurrent ECC compared to only 15.1% in caries-free children. In contrast, Praveen et al. [39] found no significant association between the presence of *S. mutans* and caries in adults aged 18 to 35. Similar results were reported by Hayes et al. (2016), who found no association between root caries experience and Mutans streptococci levels in adults over 65 years of age [40]. In the present study with older adults (65+ years old), *S. mutans* was detected in 89.7% of all participants, with similarly high prevalence in those with (94.6%) and without (85.2%) root caries. These findings suggest that while *S. mutans* is a strong predictor of caries in young children, its presence alone is not sufficient to predict disease in older populations, highlighting the importance of considering additional microbial factors such as strain-level analyses and co-association with other microorganisms [41].

Previously, our research team demonstrated in an *in vitro* study that the expression of collagen-binding proteins (Cbps) enhances the ability of *S. mutans* to adhere more strongly to collagen-rich regions of the tooth, such as dentin and root surfaces [12]. In a subsequent clinical study [10], we compared the infection levels among children with active caries lesions who were infected with either Cbp^+^ or Cbp^−^ *S. mutans*. Plaque samples collected from dentin lesions revealed that Mutans streptococci counts were significantly higher (8-fold) in children infected with Cbp⁺ *S. mutans* compared to those infected with Cbp⁻ strains. Notably, the increased colonisation rates are likely due to the ability of *S. mutans* strains harbouring the collagen-binding proteins Cnm and Cbm to adhere to oral surfaces in a sucrose-independent manner, particularly to collagen-rich substrates [42]. Building on this premise, we hypothesised that older adults, who often experience gum recession and increased root surface exposure, may be more susceptible to root caries when infected with Cbp⁺ *S. mutans* in comparison to those infected with Cbp^−^ *S. mutans*. In the present study, Cbp⁺ *S. mutans* was detected in 24.8% of the samples, aligning with existing literature that reports the presence of *cnm* and *cbm* genes in approximately 20% of *S. mutans* clinical isolates [11]. In support of our hypothesis, we observed a significant association between infection with Cbp⁺ *S. mutans* and the presence of root caries. Moreover, subjects harbouring these strains had more than 2.6 times the odds of having root caries compared to those not infected with Cpb+strains. These results contrast with a previous study that suggested Cbps may not play a significant role in the development of dental caries [43]. In fact, our findings suggest that while the general presence of *S. mutans* is not a strong predictor of root caries in older adults, infection with specific, virulent Cbp⁺ *S. mutans* strains is a strong predictor of root caries.

In accordance with previous studies that highlighted the synergistic effect of *S. mutans* and *C. albicans* co-infections in severe coronal caries in children [15,18,44], our data demonstrated that co-infection with *S. mutans* and *C. albicans* is also strongly associated with root caries in older adults. In our cohort, compared to the healthy group, subjects with root caries harboured higher levels of *Candida* spp. in saliva and in plaque, especially in plaque collected from carious root surfaces. Given that type I collagen constitutes the primary organic component of root dentin [45] and that *C. albicans* is capable of adhering to and degrading collagen [46], its presence in carious root lesions is not surprising. Moreover, the co-existence of *S. mutans and C. albicans* enhances biofilm formation and their acidogenic potential, fostering a highly cariogenic microenvironment that ultimately leads to tissue demineralisation. This effect is particularly critical in less mineralised tissues such as dentin, where the lower mineral content organic-rich composition and reduced capacity for remineralization can result in more rapid progression of caries lesions [47]. Of note, the well-established synergism of *S. mutans* and *C. albicans* is an important factor associated with caries risk. It has been shown that *C. albicans* and *S. mutans* dual-species biofilms support and protect each other under challenging conditions, such as oxidative stress [23] or following antimicrobial treatments. In particular, dual-species biofilms treated with nystatin showed reduced *S. mutans* levels and overall biofilm biomass *in vitro*, even under high-sugar conditions [48]. Thus, co-infection with *S. mutans* and *C. albicans* plays a critical role in the pathogenesis of root caries, underscoring the need to consider polymicrobial interactions in both diagnostics and therapeutic strategies.

Microbiome analysis revealed a clear segregation of bacterial community structures between saliva and dental plaque samples. This finding aligns with the well-established concept that the oral microbiome composition is site-specific [49]. However, we did not observe significant differences in microbial composition or structure between individuals with root caries and those without, suggesting that root caries may not be strongly associated with broad shifts in community diversity. Interestingly, the literature remains inconsistent regarding microbial composition in relation to caries status. While some studies have reported significant differences in both *α*- and *β*-diversity between caries-active and caries-free individuals, others have not [10,15,50,51]. Among other factors, these discrepancies are likely due to differences in the type of sample used, sample collection methods, geographical distribution and dietary habits.

In our microbiome analysis, *Veillonella parvula* appeared in significantly higher abundance in plaque collected from root surfaces compared to enamel surfaces. In a root caries rat model, Li et al. [14] found that the colonisation of *V. parvula* increased the abundance and virulence of *S. mutans* and *C. albicans*, leading to the formation of a polymicrobial biofilm with enhanced cariogenicity. Also, *V. parvula* stimulated the production of extracellular polysaccharides (EPS) by upregulating EPS-related genes (*gtfB* and *gtfC*) and promoting the proliferation of *S. mutans* and in both dual- and multi-species biofilms [14]. Additional studies are warranted to further elucidate whether the increased abundance of *V. parvula* in biofilms, particularly those on root surfaces, can serve as a potential predictor of root caries. We also found that *Capnocytophaga leadbetteri* was significantly more abundant in the roots of individuals with root caries compared to healthy controls. Although little is known about the pathogenicity of *C. leadbetteri*, a previous study has identified this anaerobic bacillus as a potential reservoir of *β*-lactam resistance genes within the oral microbiota, particularly in cases of periodontitis and haematological infections, but not in periodontally healthy individuals [52]. Acharya et al. [53] reported a higher abundance of *C. leadbetteri* in the saliva of treated and well-maintained chronic periodontitis patients compared to healthy controls with similar bleeding on probing scores, suggesting a possible link to periodontitis susceptibility. To our knowledge, this is the first report associating *C. leadbetteri* with root caries. In this study, individuals with Stage 1, 2, 3 or 4 periodontitis, as well as those with a history of periodontal disease, exhibited a higher prevalence of root caries, which could account for the observed association between *C. leadbetteri* and root caries. Further studies are warranted to explore its potential role as a biomarker for root caries.

Multiple studies have reported that birth sex influences the prevalence of root caries, although there is controversy as to whether the effect is greater in males or females [54,55]. In our study, a statistically significant association was found between birth sex and root caries status, with females showing significantly lower odds of developing root caries compared to males. This finding may be explained by differences in oral health perception and behaviour between the sexes. A review of the literature [56] reveals that males are more likely to neglect their oral health, maintain poorer oral hygiene practices and exhibit higher rates of periodontal disease, oral cancer and dental trauma. Additionally, males tend to visit the dentist less frequently and are more likely to seek dental care for acute problems rather than for preventive measures, compared to females. These behavioural differences highlight the need to consider sex-specific factors in the prevention and management of root caries.

While none of the medical conditions examined were associated with root caries in our convenience cohort, the presence of existing root surface restorations, self-reported dry mouth, use of manual toothbrushes, low frequency of flossing and severe periodontal status were associated with root caries. These findings are consistent with established caries risk factors identified in the literature [3,57]. For instance, xerostomia has been recognised as a significant risk factor for root caries, as the salivary flow is essential for neutralising acids and inhibiting bacterial growth [58]. Similarly, poor oral hygiene practices, such as infrequent flossing and reliance on manual toothbrushes, can lead to increased plaque accumulation, thereby elevating the risk of root caries [59]. Severe periodontal disease, characterised by gingival recession and attachment loss, exposes the root surface, making it more accessible to acidogenic and aciduric microorganisms that inhabit the supragingival plaque. Thus, periodontitis has been linked to a higher prevalence of root caries [60,61]. The presence of existing root surface restorations may also contribute to root caries development, as patients with previous caries experience have a higher risk of developing caries in the future [62]. Of note, caries development is time-dependent and is a result of microbial dysbiosis driven by changes in the oral environment. Thus, the observation that previous interventions (i.e. restorations) are a risk factor for root caries highlights that once microbial dysbiosis occurs, it requires increased surveillance and preventive approaches to avoid new lesions. Surprisingly, individuals wearing complete dentures exhibited a lower prevalence of root caries. We attributed this observation to the reduced number of remaining natural teeth susceptible to caries development. Taken altogether, these associations reflect the multifactorial nature of root caries and reinforce the value of comprehensive caries risk assessments as a critical tool in preventive dental care.

Collectively, this cross-sectional study provides a comprehensive evaluation of microbial, biological, dental, medical and behavioural factors associated with root caries in older adults. We showed that males, co-infection with *C. albicans* and Cbp^+^ *S. mutans* strains, elevated levels of Mutans streptococci and *Candida* spp. in plaque and saliva, presence of existing root surface restorations, dry mouth, type of toothbrush used, frequency of flossing and periodontal status may serve as potential predictors of root caries in older adults. However, due to the cross-sectional nature, caution is warranted when interpreting these findings, as causal relationships cannot be established. Cross-sectional designs capture data at a single time point and therefore cannot establish temporal sequences or directionality of associations. Additionally, as the data for this study were collected from a single centre in one country, this represents a limitation and generalisation of the findings should be interpreted with caution. Another limitation of this study is the use of a sampling-based approach for both *Candida* spp. identification and *S. mutans* Cbp status as the analysis of a limited number of colonies per specimen may have led to an underestimation of less abundant species or strains, respectively. Despite these limitations, our study provides novel insights into potential root caries predictors and highlights the need for future longitudinal studies to validate the predictive value and temporal relevance of the identified variables in root caries development.

## Supplementary Material

Supplementary Material Supplemental documents.docx

## Data Availability

The raw sequences were deposited in the Sequence Read Archive BioProject ID PRJNA1312686.
