# Supplementary material for: Co-infection with Cbp+ Streptococcus mutans and Candida albicans is associated with root caries in older adults
Source: J Oral Microbiol. 2026 May 5;18(1):2667029. doi: 10.1080/20002297.2026.2667029 (PMC13151771; doi:10.1080/20002297.2026.2667029)
Supplement: Supplementary Material — Supplemental documents.docx [file ZJOM_A_2667029_SM1963.docx]

| **Supplementary Table S1. PCR primers used for *S. mutans* detection and *cnm*/*cbm* detection** | | | | |
| --- | --- | --- | --- | --- |
| **Primer** | **Purpose** | **Sequence (5'-3')** | **Expected size (bp)** | **Reference** |
| sm479F | *S. mutans* detection | TCG CGA AAA AGA TAA ACA AAC A | 479 | 31 |
| sm479R |  | gcc cct tca cag ttg gtt ag |  | 31 |
| cnm541-F | Identification of *cnm* | AGC TGA GGT TAC TGT CGT TA | 361 | 32 |
| cnm901-R |  | CAG GAT TGT CAA CTT TAG TC |  | 32 |
| cbm-EF | Identification of *cbm* | AGC TGA AGT TAG TTA AAA CCT GCT TC | 393 | 33 |
| cbm-ER |  | TAG GAT CAT CAA CGT CAA GTA CAC GA |  | 33 |

**Supplementary Table S2:** Pairwise PERMANOVA analysis based on Euclidean distances calculated from centered log-ratio (CLR)–transformed species-level abundance data comparing healthy individuals and those with root caries.

| **Pairs** | **R^2^** | **p-value** | **p-adjusted** |
| --- | --- | --- | --- |
| Healthy enamel vs Root caries non carious root | 0.05312 | 0.001 | 0.021 |
| Healthy enamel vs Root caries enamel | 0.03388 | 0.097 | 1 |
| Healthy enamel vs Root caries | 0.07280 | 0.001 | 0.021 |
| Healthy enamel vs Healthy root | 0.05761 | 0.001 | 0.021 |
| Healthy enamel vs Root caries saliva | 0.38705 | 0.001 | 0.021 |
| Healthy enamel vs Healthy saliva | 0.41732 | 0.001 | 0.021 |
| Root caries non carious root vs Root caries enamel | 0.04844 | 0.002 | 0.042 |
| Root caries non carious root vs Root caries | 0.03311 | 0.123 | 1 |
| Root caries non carious root vs Healthy root | 0.01940 | 0.957 | 1 |
| Root caries non carious root vs Root caries saliva | 0.34607 | 0.001 | 0.021 |
| Root caries non carious root vs Healthy saliva | 0.38468 | 0.001 | 0.021 |
| Root caries enamel vs Root caries | 0.07786 | 0.001 | 0.021 |
| Root caries enamel vs Healthy root | 0.04872 | 0.001 | 0.021 |
| Root caries enamel vs Root caries saliva | 0.4136 | 0.001 | 0.021 |
| Root caries enamel vs Healthy saliva | 0.44076 | 0.001 | 0.021 |
| Root caries vs Healthy root | 0.03767 | 0.018 | 0.378 |
| Root caries vs Root caries saliva | 0.35292 | 0.001 | 0.021 |
| Root caries vs Healthy saliva | 0.38402 | 0.001 | 0.021 |
| Healthy root vs Root caries saliva | 0.36317 | 0.001 | 0.021 |
| Healthy root vs Healthy saliva | 0.40313 | 0.001 | 0.021 |
| Root caries saliva vs Healthy saliva | 0.01534 | 0.993 | 1 |
| Saliva samples vs Plaque samples | 0.31184 | 0.001 | - |
| Saliva samples vs Plaque samples (controlling for disease status – Caries, Healthy) | 0.31786 | 0.001 | - |


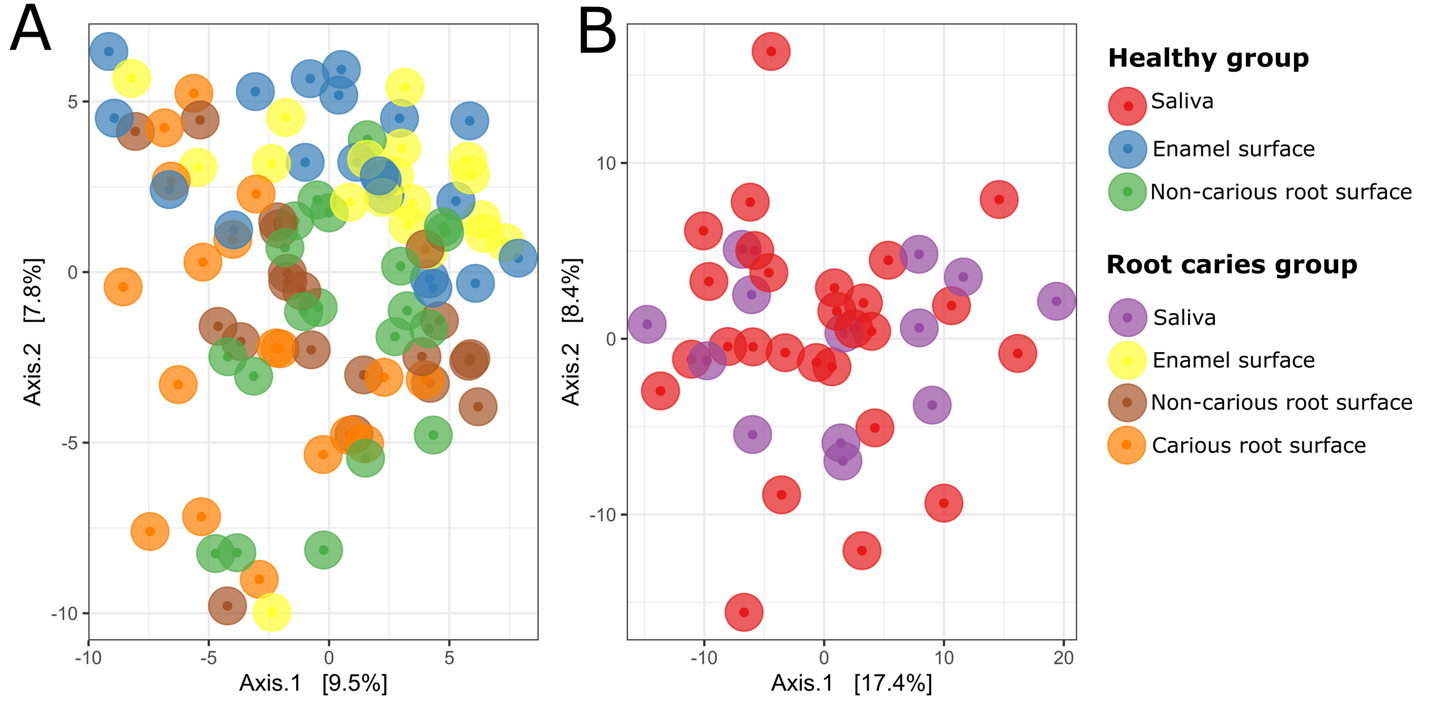


**Figure S1.** Principal coordinates analysis (PCoA) plots based on Euclidean distances calculated from centered log-ratio (CLR)–transformed species-level abundance data comparing the bacterial species community composition of saliva and plaque samples from healthy participants and those with root caries. A) Plaque samples. B) Saliva samples. Each point represents a sample, and distances between points reflect differences in overall microbial community structure. The percentages shown on Axis 1 and Axis 2 indicate the proportion of total variance in the CLR-transformed community data explained by each principal coordinate.
